# Gynecomastia-Like Hyperplasia of Axillary Ectopic Breast Tissue in a Young Female

**DOI:** 10.1155/2013/634248

**Published:** 2013-08-01

**Authors:** Joseph Shatzel, Asher Blum, Thaer Khoury, Janine Milligan, Joseph J. Skitzki

**Affiliations:** ^1^Department of Surgical Oncology, Roswell Park Cancer Institute, Buffalo, NY 14263, USA; ^2^Department of Pathology and Laboratory Medicine, Roswell Park Cancer Institute, Buffalo, NY 14263, USA; ^3^Department of Diagnostic Radiology, Roswell Park Cancer Institute, Buffalo, NY 14263, USA

## Abstract

Gynecomastia-like hyperplasia of orthotopic female breast tissue is a rare entity. We present the singularly unique case of a 22-year-old female who presented with a small axillary mass subsequently discovered to be a discrete deposit of ectopic breast tissue with gynecomastia-like hyperplasia. This case highlights the etiology, variable presentation, and evaluation of ectopic breast tissue.

## 1. Introduction

While malignancy must be included in the differential diagnosis of axillary masses, there are a variety of benign pathologies which must be considered. One entity is ectopic breast tissue which is found in approximately 1% of the population and may also be a source for neoplasm [1, 2]. Even more uncommon is gynecomastia-like hyperplasia which is an extremely rare finding in orthotopic female breast tissue [3]. We present the unique case of this unusual histology arising from ectopic breast tissue in a female.

## 2. Clinical History

A healthy, nulliparous 22-year-old female was referred to our clinic for the complaint of a nonresolving tender right axillary mass. After a month of noticing a small, tender lump in her right axilla, she was given a course of antibiotics. The nodule did not change and after an additional month of observation, she was referred to our clinic for further evaluation.

The patient denied any prior history of trauma, infection, breast mass, or previous lymphadenopathy. She regularly shaves her axillary region and uses underarm antiperspirant, with no recent change in brands. She reported that the size of the mass had remained constant and was tender to palpation with no overlying erythema, fluctuance, or other skin changes. Her symptoms did not vary with her menstrual cycle and were not associated with oral contraceptive use which was initiated a few years prior. The patient denied any history of fever, chills, night sweats, or other signs of systemic infection. Lastly, she denied any history of superficial soft tissue infections, rashes, travel, or exposure to cats. A complete review of systems was otherwise negative.

On exam, she was found to have a single, tender, mobile, subcutaneous mass approximately 1.5 cm in diameter, with no erythema, induration, or overlying skin changes. The mass appeared to be truly subcutaneous at the interface of skin between the superior axilla and the medial arm. There was no palpable lymphadenopathy in the nodal basins. Breast exam did not reveal any palpable masses or abnormalities.

## 3. Materials and Methods

Utilizing the Philips iU22 ultrasound system (Philips Healthcare, Andover, MA) and a 12.5 linear array ultrasound probe, generous survey scanning of the right axilla was performed. Gray scale and color Doppler images of the right axilla in the area of palpable abnormality were obtained. 

 Formalin-fixed paraffin-embedded tissue was obtained from excision. It was cut to 5 *μ*M, placed on charged slides, and stained with hematoxylin and eosin using Sakura Tissue-Tek Prisma stainer (Torrance, CA).

## 4. Results

**Figure 1:**
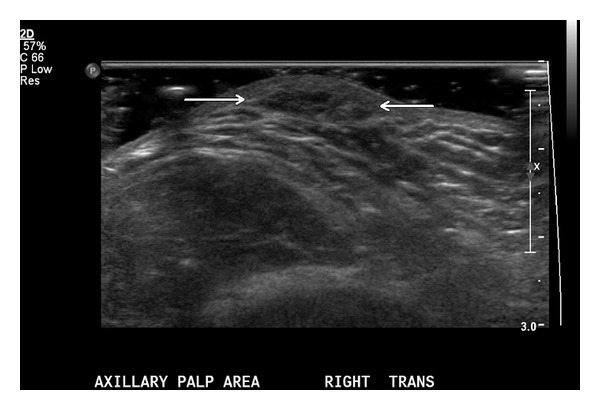
Representative ultrasound imaging of the right axilla demonstrates the area of palpable abnormality an approximately 1.2 cm oval, hypoechoic solid-appearing lesion. No capsule or posterior acoustic enhancement was evident. No vascularity was identified within the lesion on color Doppler interrogation and the adjacent vessels within the axilla were compressible and patent.

Gray scale and color Doppler imaging of the right axilla was performed (Figure 1). In the area of palpable abnormality, an approximately 1.2 cm oval, hypoechoic solid-appearing lesion was identified. No capsule or posterior acoustic enhancement was present. No vascularity was identified within the lesion on color Doppler interrogation. The adjacent vessels within the axilla were compressible and patent. No axillary lymph nodes were identified. Differential diagnosis included benign entities such as lipoma, a focus of lipohypertrophy, fat necrosis/fat trauma, or focal cellulitis. The patient requested removal of the mass, as she continued to be symptomatic and desired a definitive diagnosis. Therefore, she had an uncomplicated excisional biopsy performed under local anesthetic. 

**Figure 2:**
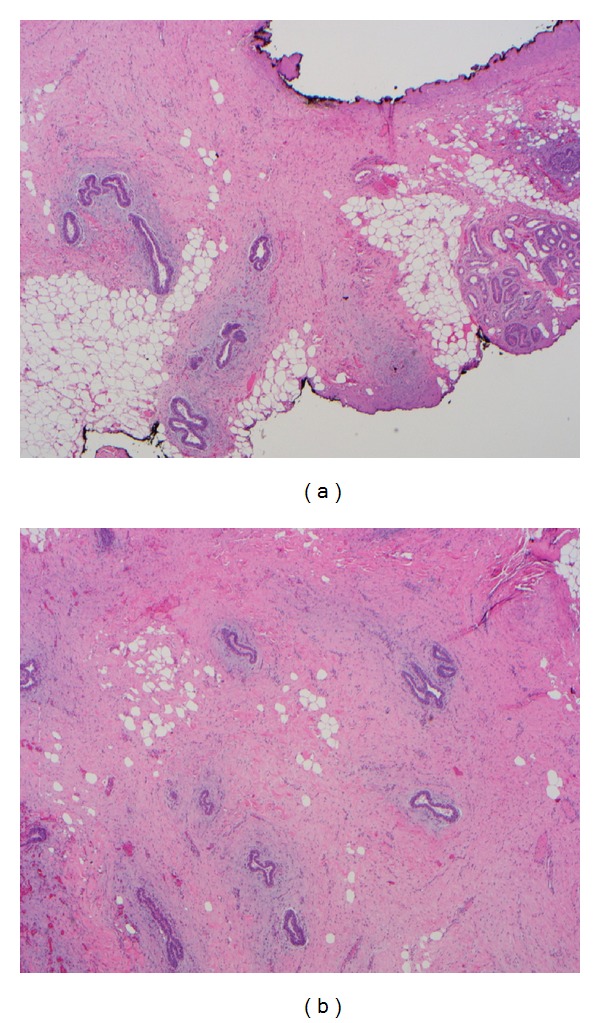
(a) Histopathology examination revealed an ill-defined lesion composed of irregularly proliferating mammary ducts with no lobules. The surrounding stroma was reactive and exaggerated. (b) The lesion extended adjacent to the axillary skin adnexa. Evenly distributed irregular ducts with periductal stromal proliferation were present, without atypia, consistent with a diagnosis of gynecomastia-like hyperplasia within an ectopic mammary tissue deposit.

A 1.5 cm × 1.2 cm × 0.5 cm sample was sent for pathologic evaluation. The lesion was ill defined and composed of irregularly proliferating mammary ducts with no lobules. The surrounding stroma was reactive and exaggerated (Figure 2(a)). The lesion extended adjacent to the axillary skin adnexa (Figure 2(b)). Evenly distributed irregular ducts with periductal stromal proliferation were present, without atypia, consistent with a diagnosis of ectopic mammary tissue. Therefore, the final diagnosis was ectopic mammary tissue of the subcutaneous tissue with gynecomastia-like hyperplasia. 

## 5. Discussion

While the exact incidence of ectopic breast tissue is unclear, it is generally believed to be about 1% in the general population [4, 5], although incidences between 0.2% to 6% have been cited in the literature [2, 4, 6]. The incidences appear to be higher in Asian [4] and Native American women [2]. In utero, ectopic breast tissue develops by the 4th-5th week of human embryonic development as two mammary ridges extend from the axilla to the groin [4, 7]. Incomplete regression of these mammary ridges during embryogenesis results in ectopic mammary tissue [6]. Therefore, ectopic breast tissue generally occurs ventrally along the embryonic milk line. The axilla and vulva are the most common sites [8], but ectopic breast tissue has been reported at numerous other sites including the face, neck, shoulder, flank, hip, thigh, anus, and foot [1, 9–11]. Axillary ectopic breast tissue presents bilaterally in up to 30% of cases, and its discovery should alert for the potential presence of other sites of ectopic tissue [7].

Currently, a standardized terminology within the literature to describe the various manifestations of ectopic breast tissue is lacking. Examples range from simple glandular tissue to complete nipple and areola complexes being present. The initial classification scheme was described by Kajava in 1915 [12]. The terms ectopic, or aberrant breast tissue, polythelia, and polymastia have all been used with frequency in the literature, and at times indistinguishably. Ectopic breast tissue often represents a diagnostic challenge. Clinical signs such as correspondence of symptoms with menstrual periods, puberty, and pregnancy or even signs of lactation can aid in diagnosis [13]. 

A thorough history and physical exam is needed when working up an axillary mass including examination of the breast and thyroid. Chest X-ray and bilateral mammography have been suggested for cases where a direct cause cannot be determined by history and physical examination [14]. Ultimately, a tissue diagnosis is needed to distinguish between benign and malignant disease. Our patient's diagnosis of gynecomastia-like hyperplasia arising from ectopic breast tissue was made only after pathologic evaluation of the biopsied tissue.

Our patient's pathology was unique in several regards. Gynecomastia is a term generally reserved for male pathology. The term “gynecomastia-like hyperplasia” was first described by Rosen in 1997, to describe extremely rare proliferative lesions of the female breast that are indistinguishable from florid gynecomastia [15]. Likewise, Umlas highlighted the rarity of gynecomastia-like hyperplasia when he examined the pathology of 1242 breast excisions performed over a 26 month period [3]. Only four patients out of the twelve hundred were found to have gynecomastia-like lesions. Accordingly, we could find no previous reports of ectopic gynecomastia-like lesions arising from ectopic breast tissue in a female, making this the first reported case. 

In summary, ectopic breast tissue is an increasingly recognized cause of axillary masses. It can present as an acutely tender axillary mass and may be considered in the differential if a thorough history and physical examination does not reveal an obvious cause. A tissue biopsy is required for diagnosis as ectopic breast tissue can undergo the same malignant changes as normal breast tissue. Lastly, gynecomastia-like hyperplasia of the female breast is a rare entity and, based on this case, can present in ectopic breast tissue.
